# Development of the Japanese version of the Visual Discomfort Scale

**DOI:** 10.1371/journal.pone.0191094

**Published:** 2018-01-11

**Authors:** Shu Imaizumi, Shinichi Koyama, Yoshihiko Tanno

**Affiliations:** 1 Graduate School of Arts and Sciences, The University of Tokyo, Tokyo, Japan; 2 Japan Society for the Promotion of Science, Tokyo, Japan; 3 Graduate School of Engineering, Chiba University, Chiba, Japan; Eberhard-Karls-Universitat Tubingen Medizinische Fakultat, GERMANY

## Abstract

**Background:**

Visual stimuli, such as stripes and texts, can induce “visual discomfort” including perceptual and somatic symptoms. Individuals reporting high levels of visual discomfort might experience migraine headache and may have reduced reading efficiency due to visual perceptual difficulties. This study aimed to develop and validate the Japanese version of the Visual Discomfort Scale, which measures proneness to visual discomfort.

**Methods and results:**

In Survey 1, 428 adults completed the Japanese version and a questionnaire assessing migraine morbidity. Rasch analysis revealed that the Japanese version is a unidimensional scale with a high amount of unexplained variance due to random noise rather than another dimension, and has high person and item reliabilities. Participants with migraine exhibited high scores in the Japanese version, indicating the construct validity of the scale. Survey 2 with 118 adults revealed a strong test-retest correlation for the Japanese version, indicating the stability of the scale.

**Conclusion:**

The Japanese version of the Visual Discomfort Scale is a sufficiently reliable and valid scale for assessing visual discomfort, although its unidimensionality leaves room for further improvements.

## Introduction

Visual stimuli, such as high-contrast repetitive stripes, printed text, and complex images, can induce “visual discomfort” that includes perceptual distortions (e.g., shimmer, glare), somatic symptoms (e.g., eyestrain, headache), and self-reported discomfort and aversiveness to view [1–3]. Moreover, visual discomfort can occur when reading texts, for example, letters moving, flickering, or disappearing, and consequently can hinder reading comprehension [4, 5]. The origin of visual discomfort is partly in the sensory and neuronal responses to the physical properties of visual stimuli; for example, their spatial frequency [3, 6–8] and Fourier energy distribution [9–12]. Another origin can be attributed to individual clinical conditions such as photosensitive epilepsy [3, 6, 13, 14] and migraine headache [3, 15–21], whose perceptual and neuronal hyperactivities have been investigated. Notably, even individuals without certain clinical conditions can experience visual discomfort [9–12] with substantial individual differences [22–24].

Previous studies have examined visual discomfort and its variation in normal and clinical populations based on behavior (e.g., [9]), electrophysiology (e.g., [8]), and brain imaging [18, 25–27]. On the contrary, psychometric scales can also be useful when investigating perceptual inter-individual variability. For example, questionnaires on multi-sensory sensitivity [28] and face recognition [29] have been used to investigate their individual differences in both clinical and non-clinical populations. A psychometric approach may have several advantages; questionnaire surveys with larger samples can enable the investigation of the effects of demographic and psychological factors on the constructs of interest. Moreover, questionnaires can serve as a control measure and a variable explaining the behavioral and physiological responses in experimental studies.

Conlon et al. [22] developed the Visual Discomfort Scale (VDS) that consists of 23 items rated on a four-point scale to measure the tendency to experience visual discomfort on a daily basis. The developers confirmed the unidimensional structure of the VDS using the Rasch modeling analysis [30]. To date, studies using the VDS have elucidated perceptual mechanisms underlying visual discomfort. For example, individuals with high VDS scores can show declines in contrast sensitivity [7], global motion sensitivity [31], accommodation [32, 33], visual-search performance [34, 35], and reading comprehension [36]. In terms of methodological benefits of the VDS, besides the comparison between individuals with high and low proneness, the VDS has also been used to control the participants’ proneness to visual discomfort and potential confounders due to various altered visual processing [37–41], as mentioned above. Moreover, given that the VDS, which measures chronic symptoms of visual discomfort, can also predict acute symptoms [42] and assess academic problems that may occur due to visual discomfort [43], the VDS may be useful not only for empirical studies but also for clinical and educational assessments. However, to our knowledge, no published study has developed and validated the translated version of the VDS. Previous studies in non-English-speaking countries have used the VDS, but have not mentioned the validation of its translated versions [37, 39–41, 44].

The current study aimed to develop a Japanese version of the VDS and to examine its reliability and validity. In Survey 1, we translated the VDS into Japanese and subsequently conducted an in-person survey with Japanese adults. The Rasch model analysis was used to examine the fit of the Japanese VDS to a unidimensional measurement structure similar to the original version [22], and to assess its internal consistency. To test the construct validity of the Japanese VDS, we examined the relationship of the VDS score to the morbidity of migraine headache. People with migraine are prone to visual discomfort even in the interictal period [15, 17–21]. Thus, if the Japanese VDS has sufficient construct validity, it is expected that participants with migraine will score higher on the VDS than those without any primary headache will (i.e., known-groups validity). In Survey 2, we conducted an online survey recruiting another sample of Japanese adults to examine the stability (i.e., test-retest reliability) of the Japanese VDS.

## Survey 1: Translation, reliability, and construct validity

This survey aimed to develop a Japanese translation of the VDS, and to confirm its reliability and validity. First, we collected data from Japanese adults and analyzed whether the Japanese VDS has a unidimensional measurement structure using Rasch modeling, according to that observed in the original version [22]. Second, using the Rasch analysis, we examined the reliability of the Japanese VDS. Finally, we analyzed the construct validity of the Japanese VDS by testing whether individuals with migraine headache, which are likely to accompany reports of visual discomfort, would score high on the Japanese VDS.

### Materials and methods

#### Ethics statement

The current study was approved by the local ethics committee of the Graduate School of Engineering, Chiba University, and was conducted in accordance with the Declaration of Helsinki. Prior to the survey, participants were informed that participation was voluntary and that they could quit at any time. Written and electronic informed consent was provided by each participant in Survey 1 and 2, respectively.

#### Translation of the Visual Discomfort Scale

Before the commencement of the current study, the first author acquired permission to translate the VDS into Japanese from the first author of the original version [22]. The present first author and two professional translators individually translated the original English VDS into Japanese. The present first author then compared these translated versions and developed the Japanese VDS. The back-translation was conducted by two other professional bilingual translators, who did not know about the study’s purpose and the original VDS. After two other non-professional bilinguals and the present authors individually confirmed consistency between the original and back-translated VDS versions, the present first author added some minor modifications to the format and completed developing a tentative version of the Japanese VDS. Two Japanese female undergraduates completed the tentative version and confirmed its comprehensibility. Finally, we decided to use this as the final version of the Japanese VDS in the surveys that followed.

#### Participants and procedures

A total of 453 Japanese students from two colleges located in Tokyo and Chiba (both in Eastern Japan) voluntarily participated in this survey and received no compensation. Printed questionnaires, which included an ethics statement, consent form, and demographic questions (sex, age, and visual acuity), followed by psychometric measures (see Measures), were distributed to the participants by visiting some classrooms and laboratories. A total of 25 of the participants, who provided more than one missing response, were excluded from the analysis. We finally analyzed data collected from 428 participants (247 females; mean age = 23.21 years, standard deviation [*SD*] = 6.77 years, range = 18 to 60 years). All participants reported that they had normal or corrected-to-normal visual acuity. Our sample did not greatly differ from that used in the study in which the original VDS was developed [22], which comprised 515 students in Australia, whose age ranged from 16 to 48 years (mean age and sex ratio unavailable).

#### Measures

The Japanese VDS and a migraine screening questionnaire were completed by the participants.

The VDS is a Rasch-based unidimensional scale consisting of 23 items [22]. Each VDS item (Table 1; see S1 File for the Japanese version) assesses the frequency of everyday experiences regarding the perceptual and somatic aspects of visual discomfort induced when reading texts and when observing striped patterns and lights. The items are answered using a four-point scale with 0 (“Event never occurs”), 1 (“Occasionally. A couple of times a year”), 2 (“Often. Every few weeks”), and 3 (“Almost always”). The sum of the item scores serves as the VDS scale score that ranges from 0 to 69, according to the original version and those reported in previous studies [26, 32, 43, 45–47]. There are no reverse-scored items.

**Table 1:** Rasch item measures, fit statistics, and latent principal component structure of the Japanese version of the Visual Discomfort Scale in Survey 1.

	Location (standard error)	Infit mean square (Zstd)	Outfit mean square (Zstd)	First contrast loading	Second contrast loading
1. Do your eyes ever feel watery, red, sore, strained, tired, dry, gritty, or do you rub them a lot, when viewing a striped pattern?	-0.60 (0.07)	1.07 (1.00)	1.29 (3.10)	0.02	**0.35**
2. Do your eyes ever feel watery, red, sore, strained, tired, dry or gritty, after you have been reading a newspaper or magazine with clear print?	-0.27 (0.08)	0.86 (-1.90)	0.81 (-2.10)	0.18	**0.58**
3. Do your eyes ever feel watery, red, sore, strained, tired, dry or gritty, when working under fluorescent lights?	-0.98 (0.07)	0.90 (-1.50)	0.93 (-0.90)	0.04	**0.54**
4. How often do you get a headache when working under fluorescent lights?	0.18 (0.08)	1.09 (1.00)	1.12 (1.00)	0.08	**0.45**
5. Do you ever get a headache from reading a newspaper or magazine with clear print?	0.82 (0.10)	1.02 (0.20)	0.85 (-1.00)	0.03	**0.52**
6. When reading, do you ever unintentionally re-read the same words in a line of text?	-0.88 (0.07)	0.94 (-0.90)	1.01 (0.10)	**0.70**	-0.25
7. Do you have to use a pencil or your finger to keep from losing your place when reading a page of text in a novel or magazine?	-0.14 (0.08)	1.28 (3.40)	1.42 (3.60)	**0.33**	-0.06
8. When reading do you ever unintentionally re-read the same line?	-0.85 (0.07)	0.95 (-0.80)	1.02 (0.30)	**0.69**	-0.21
9. When reading do you ever have to squint to keep the words on a page of clear text from going blurry or out of focus?	-0.16 (0.08)	1.14 (1.80)	1.03 (0.30)	-0.32	0.08
10. When reading, do the words on a page of clear text ever appear to fade into the background then reappear?	0.99 (0.11)	1.21 (1.90)	1.06 (0.40)	-0.02	-0.13
11. Do the letters on a page of clear text ever go blurry when you are reading?	0.03 (0.08)	0.95 (-0.60)	0.90 (-0.90)	-0.25	0.01
12. Do the letters on a page ever appear as a double image when you are reading?	0.22 (0.09)	1.01 (0.10)	0.95 (-0.30)	-0.40	-0.13
13. When reading, do the words on the page ever begin to move or float?	0.55 (0.09)	1.13 (1.40)	0.95 (-0.30)	-0.23	-0.35
When reading, do you ever have difficulty keeping the words on the page of clear text in focus?	-0.34 (0.07)	0.85 (-2.10)	0.81 (-2.10)	-0.06	-0.35
15. When you are reading a page that consists of black print on white background, does the background ever appear to overtake the letters making them hard to read?	1.23 (0.12)	1.16 (1.40)	0.72 (-1.60)	-0.29	-0.38
16. When reading black print on a white background, do you ever have to move the page around, or continually blink to avoid glare which seems to come from the background?	0.18 (0.08)	1.00 (0.10)	0.86 (-1.20)	-0.39	-0.02
17. Do you ever have difficulty seeing more than one or two words on a line in focus?	0.23 (0.09)	1.03 (0.40)	0.90 (-0.80)	-0.02	-0.32
18. Do you ever have difficulty reading the words on a page because they begin to flicker or shimmer?	-0.41 (0.07)	0.73 (-4.00)	0.73 (-3.20)	-0.24	-0.01
19. When reading under fluorescent lights or in bright sunlight, does the glare from the bright white glossy pages cause you to continually move the page around so that you can see the words clearly?	-0.86 (0.07)	1.31 (4.20)	1.26 (3.10)	-0.26	0.06
20. Do you have to move your eyes around the page, or continually blink or rub your eyes to keep the text easy to see when you are reading?	-0.06 (0.08)	0.89 (-1.40)	0.82 (-1.70)	-0.39	0.02
21. Does the white background behind the text ever appear to move, flicker, or shimmer making the letters hard to read?	0.83 (0.10)	0.96 (-0.40)	0.71 (-2.00)	-0.38	-0.15
22. When reading, do the words or letters in the words ever appear to spread apart?	1.01 (0.11)	1.14 (1.30)	0.89 (-0.60)	0.07	-0.21
23. As a result of any of the above difficulties, do you find reading a slow task?	-0.73 (0.07)	1.16 (2.20)	1.09 (1.10)	**0.38**	-0.31

Zstd: z-standardized statistic. Bold fonts indicate contrast loading larger than 0.30.

The migraine screening questionnaire included 18 items on the occurrence of primary headaches and their duration, characteristics, and accompanying symptoms (see S2 File for details), which were designed to conform to the third edition (beta) of the International Classification of Headache Disorders [48, 49]. Responses to the migraine screening questionnaire were evaluated according to the criteria of the International Classification of Headache Disorders. The criteria for migraine without aura were as follows (aura refers to transient visual, sensory, or motor disturbance before a migraine attack). The experience of five or more migraine attacks during one’s lifetime was required. Each migraine attack was required to last for 4–72 hours and be accompanied by at least two of the following characteristics: unilateral location, pulsating quality, moderate or severe intensity, and aggravation by physical routine activity (e.g., walking). In addition, the attacks were required to be associated with at least one of the following symptoms: nausea and/or vomiting, and photophobia and/or phonophobia. Participants who met all the above criteria were classified into the Migraine category, while those who satisfied all but one of the criteria were classified into the Probable Migraine category, by reference to the International Classification of Headache Disorders and previous studies (e.g., [50, 51]). Based on this questionnaire, participants were classified into four categories: Headache-free (i.e., never experienced a primary headache; number [*N*] = 130), Migraine (*N* = 74), Probable Migraine (*N* = 144), and Others (i.e., experienced primary headache, but neither classified as migraine nor probable migraine; *N* = 80). Characteristics of the participants’ (probable) migraine headaches have been summarized in S3 File.

As migraine with visual aura (i.e., scintillating scotoma) can be associated with altered functional and anatomical alterations in visual cortices [52, 53], one might want to know about the potential effect of visual aura on the participants in the categories “Migraine” and “Probable Migraine,” all of whom were classified as migraine *without* aura. At the same time, we had to check whether there was a confounding effect of potential visual aura experience on the variables of interest. The migraine screening questionnaire for exploratory purposes includes an item about the occurrence of visual aura using a dichotomous response. Therefore, we could compare the participants with (probable) migraine *without* aura, who did and did not have an experience of visual aura, although this item did not ask about aura in detail and was not intended to formally classify as migraine *with* aura. Consequently, 12 participants each from the Migraine and Probable Migraine categories reported that they experienced visual aura at least once in their lifetime. Thus, our preliminary analysis using two-tailed *t*-tests examined differences in age and VDS score between participants with and without experience of visual aura, separately for the Migraine and Probable Migraine categories. We found no effects of visual aura for both Migraine (age: *t*(72) = 0.14, *p* = 0.89, Cohen’s *d* = 0.04; VDS: *t*(13.14) = -0.31, *p* = 0.76, *d* = 0.13) and Probable Migraine categories (age: *t*(142) = 1.16, *p* = 0.25, *d* = 0.35; VDS: *t*(142) = 0.38, *p* = 0.71, *d* = 0.11). Moreover, the chi-square test revealed no relationship between visual aura and sex for both headache categories (Migraine: *χ*^2^(1) = 1.94, *p* = 0.16, *φ* = 0.16; Probable Migraine: *χ*^2^(1) = 1.64, *p* = 0.20, *φ* = 0.11). These were consistent with previous studies that have investigated visual characteristics in migraine by comparing whether visual aura accompanies them or not, but found no differences in VDS scores [26, 46, 47, 54] and perceptual performances such as contrast sensitivity [21, 55]. Therefore, we decided not to discriminate participants with aura from those without aura in the following analyses.

Although the psychometric properties of the migraine screening questionnaire have not been evaluated and questionnaire-based screening may be quasi-valid in terms of a clinical diagnosis, our previous studies have partially validated this questionnaire. For instance, our epidemiological study in Japan using this questionnaire has revealed the prevalence of migraine and its triggering factors [56]. Importantly, our experimental studies in Japan have successfully differentiated the visual [20, 57, 58] and visuo-vestibular [59] perceptual characteristics in people with migraine from those in people without migraine classified by this questionnaire. The current study also aimed to investigate the perceptual characteristics in terms of visual discomfort, and thus adopted the migraine screening questionnaire.

#### Data analysis

According to the development study of the original version [22], the Rasch model analysis was performed using Winsteps 3.92.1 [60], to examine how well individual items of the Japanese VDS fit a unidimensional model for measuring visual discomfort. Andrich rating scale model [61] was adopted because all items shared the same polytomous response structure [62, 63]. The Rasch analysis procedure followed the recent guidelines [62–65]. First, we assessed whether the four response categories of the VDS were evenly ordered by checking a continuous ordering of the thresholds, which are points at which two adjacent category probability curves cross. Disordering of the thresholds occurs when categories are underused, ambiguously defined, or hard for respondents to distinguish [66]. Measures indicating location on the unidimensional latent variable, expressed in logits, were calculated for each person and item. Infit and outfit statistics for each item were used as indices of the fit to the Rasch model. Infit (i.e., inlier-pattern-sensitive fit statistic) is based on the chi-square statistic weighted using model variance, while outfit is based on the conventional chi-square statistic and more sensitive to outliers. Since infit and outfit mean-squares indicate the amount of distortion of the measurement scale and their expected values are close to one, values less than one indicate an overfit to the Rasch model, while those more than one indicate an underfit to the model. Mean-squares within the range from 0.70 to 1.30 reflect that the item fits well to the unidimensional model for measurement [63, 65]. We adopted this criterion while there has been a more lenient criterion between 0.50 and 1.50 has also been acceptable for considering unidimensional measurement [62, 67]. Infit and outfit z-standardized statistics (Zstds, standardized *t*-statistics being reported with infinite degrees of freedom) indicate statistical significance of the mean-squares occurring by chance, based on the assumption that the data fit the Rasch model. A Zstd less than zero indicates overfit, while that more than zero indicates underfit. When we obtained the acceptable infit and/or outfit mean-squares, their Zstds could be ignored because mean-squares close to one indicate little distortion of the measurement scale, regardless of the Zstd [62]. The unidimensionality of the scale was also assessed by a principal component analysis (PCA) of the residuals based on the amount of raw variance explained by the measure and the eigenvalue of the unexplained variance in the first contrast.

Furthermore, we analyzed the person or item reliability based on the Rasch modeling. The Rasch reliability indicates reproducibility; high person- or item-reliability means the high probability that persons or items with high estimated measures show high actual measures than persons or items with low estimated measures do. More specifically, person reliability can serve as an indicator of reproducibility of person ordering that can be expected if this sample of persons were given another set of items measuring the same construct [68], and item reliability can serve as an indicator of reproducibility of the items’ hierarchy and/or given scores to each item if the same items were given to another sample with comparable characteristics [64]. We also reported the Cronbach’s alpha, a traditional measure of internal consistency, in order to compare the findings with those of previous studies and with those of our Survey 2. Targeting, referring to how well the item difficulty matches with the abilities of the study sample, was assessed by the differential between person and item mean location measures [65]. Differential item functioning (DIF), which indicates that one subgroup of a sample is scoring different from the other subgroup on an item [65], was checked across three subgroups: sex (male or female), age (younger than the median 21 years or not), and headache (Headache-free, Migraine, Probable Migraine, or Others). DIFs for sex and age were assessed by subgroup differentials of item measure in logits, while DIF for headache was assessed by the chi-square test where the null hypothesis was that the item had no overall DIF across subgroups [62].

We tested known-groups validity by analyzing the effect of migraine headache on the Japanese VDS. A one-way analysis of variance on the VDS score was performed to compare between four participant categories: Headache-free, Migraine, Probable Migraine, and Others. If the Japanese VDS has sufficient known-groups validity, Migraine participants would score higher on the VDS than the Headache-free, Probable Migraine, and Others participants would. However, we could also expect that the difference in the VDS score between Migraine and Probable Migraine might be small or null due to the similarity of their symptoms. To test for any effect of sex and age on the VDS and primary headache properties, unpaired two-tailed *t*-test on the VDS score comparing sexes and Pearson’s zero-order correlation analysis between age and VDS were performed. In addition, chi-square tests were performed to analyze the relation between sex and headache categories, and a one-way analysis of variance was performed on age with the headache categories as a factor. If there were any sex and age effects, we performed an analysis of covariance (ANCOVA) on the VDS score with the headache categories as a factor and with the sex and/or age as covariates only when the data satisfied the assumption of the parallelism of regressions (i.e., absence of interaction between independent variable and covariate) and the significance of regression by covariate. In the current study, statistical analyses, except for Rasch modeling, were performed using SPSS 24.0 (IBM Corporation, Armonk, New York). The significance level was set at *p* < 0.05.

### Results and discussion

#### Rasch analysis

**Fig 1:**
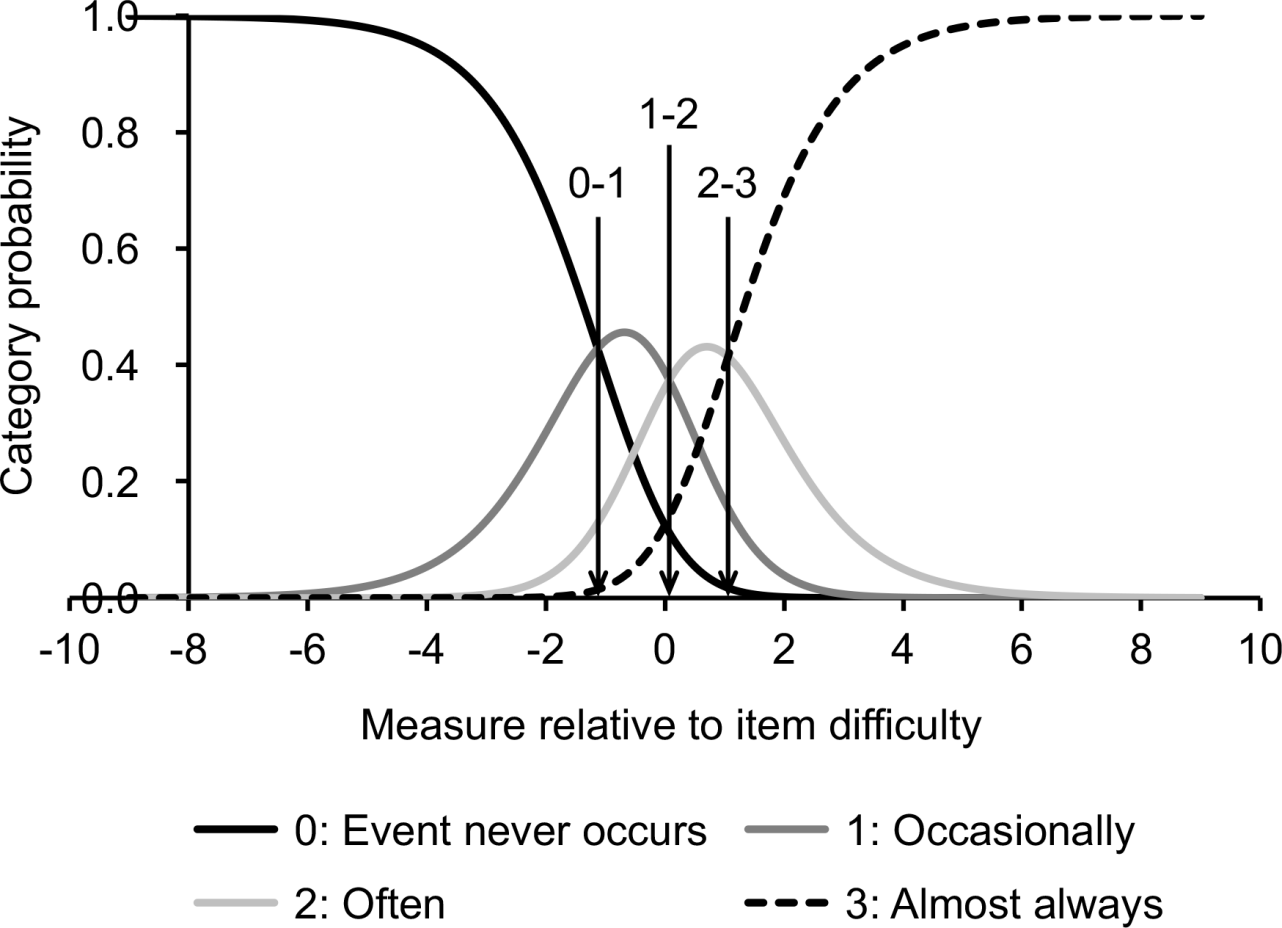
Category probability curves for the Japanese version of the Visual Discomfort Scale. The four curves show ordered response categories. Arrows indicate three thresholds for the four categories.

**Fig 2:**
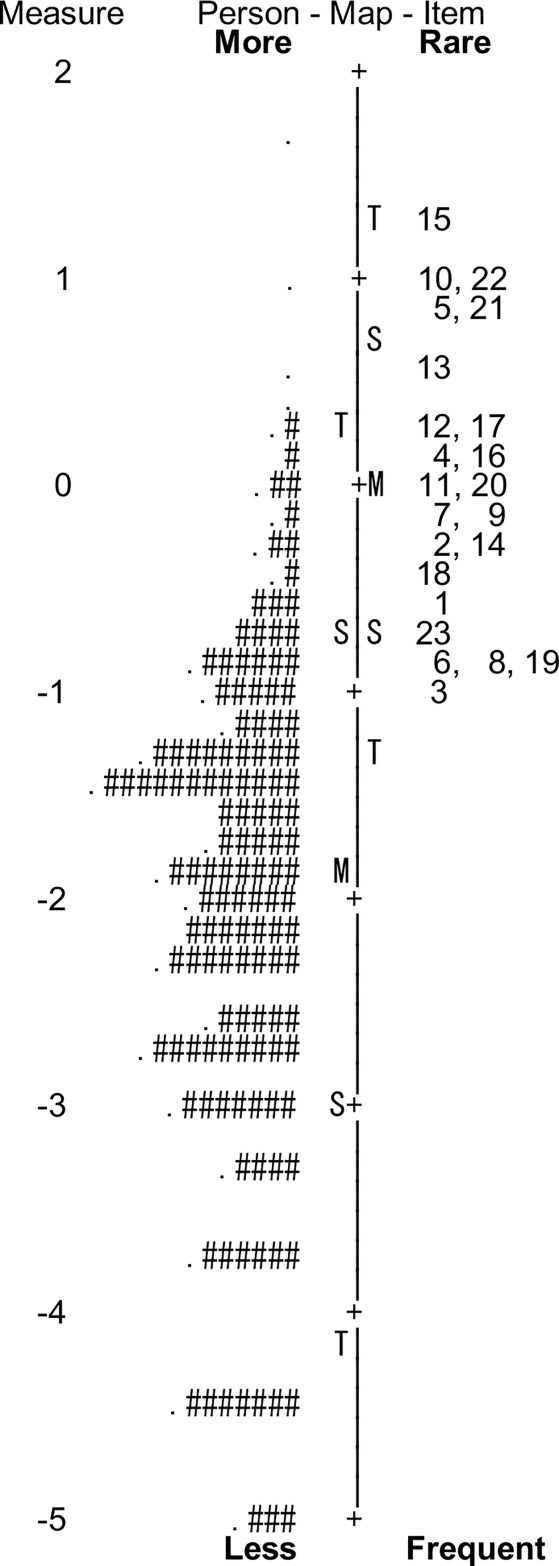
Person-item map of the Japanese version of the Visual Discomfort Scale. Person locations are displayed on the left column, while item locations are displayed on the right. Each hash represents three participants, and each period represents one or two participants. Numbers on the right column correspond to the item numbers of the Visual Discomfort Scale. M: mean; S: one standard deviation from the mean; T: two standard deviations from the mean.

There was no disordering of thresholds of the four-response categories of the Japanese VDS (Fig 1), suggesting that all response categories were understood, distinguished, and evenly endorsed by the participants. The Rasch statistics for the items and persons have been reported in Tables 2 and 3 (see S4 File for the person statistics without minimum extreme). Descriptive statistics have been presented in Table 4. Location measures for persons and items have been summarized in the Wright’s person-item map (Fig 2). The Rasch modeling analysis provided the fit statistics indicating how well each item fit to the unidimensional model of the Japanese VDS (Table 1). As a whole, items were well fitted to the model; averaged infit mean-square was 1.03 and averaged outfit mean-square was 0.96 (Table 2). Specifically, infit and outfit mean-squares were in between 0.70 and 1.30, which is the criterion range for considering that the item fits well to the model [63, 65], except for the item 7, whose outfit mean-square was 1.42, and the item 19, whose infit mean-square was 1.31 (Table 1). As another index of the unidimensionality, the PCA revealed that 38.1% of the raw variance was explained by the measures. This was below the criterion of 50.0% for unidimensionality of the scale [65]. Moreover, the eigenvalues of the unexplained variance in the first and second contrast were 2.31 and 2.03 respectively, which exceeded the cutoff of 2.00 [65]. These suggest that the Japanese VDS has a low degree of unidimensionality and that there were other underlying dimensions in the residuals. We thus examined the presence of multidimensionality in terms of the correlation between item clusters within each PCA contrast (i.e., latent dimension) [62, 64]. The items were divided into three item clusters according to loadings on each of the first and second contrast. For the first contrast, cluster 1 included items 6 and 8; cluster 2 included items 23, 7, 2, 4, 22, 3, 5, 1, 17, 10, and 14; and cluster 3 included items 13, 18, 11, 19, 15, 9, 21, 20, 16, and 12. For the second contrast, cluster 1 included item 2, 3, 5, 4, and 1; cluster 2 included items 9, 19, 20, 11, 18, 16, 7, 12, and 10; and cluster 3 included items 21, 22, 8, 6, 23, 17, 14, 13, and 15. Clusters and items were numbered in the order of contrast loading (contrast loadings of items are shown in Table 1). Each person was measured on each item cluster for each contrast. These measures for each item cluster were correlated for each pair of clusters. The correlation was reported as the disattenuated Pearson correlation coefficient, which removed a standard error of measurement for each item cluster. If the disattenuated correlation coefficient approaches 1.00, we can consider the pair of item clusters to measure the same construct [62]. The disattenuated correlation coefficients were 0.94 to 1.00 for the first contrast and 0.88 to 1.00 for the second contrast. This suggests that the item clusters defined by two latent dimensions (i.e., PCA contrast) indeed measure the same construct. Given the low degree of raw variance explained by the measures, it can be said that the Japanese VDS has unidimensionality, but there is a high amount of unexplained variance, which is considered as random noise.

**Table 2:** Rasch summary statistics for 23 items of the Japanese version of the Visual Discomfort Scale.

	Mean	Population *SD*	Sample *SD*	Min	Max
Total score	234.30	98.60	100.80	83	406
Location (standard error)	0.00 (0.08)	0.65 (0.02)	0.67 (0.02)	-0.98 (0.07)	1.23 (0.12)
Infit mean-square (Zstd)	1.03 (0.30)	0.14 (1.80)	0.14 (1.90)	0.73 (-4.00)	1.31 (4.20)
Outfit mean-square (Zstd)	0.96 (-0.30)	0.18 (1.70)	0.19 (1.70)	0.71 (-3.20)	1.42 (3.60)
**Real:** *RMSE* = 0.09, adjusted *SD* = 0.65, separation = 7.32, item reliability = 0.98					
**Model-based:** *RMSE* = 0.09, adjusted *SD* = 0.65, separation = 7.63, item reliability = 0.98					
Standard error of item mean = 0.14, Correlation between item raw scores and measures = -0.99					

*SD*: standard deviation; Zstd: z-standardized statistic; *RMSE*: root mean square error

**Table 3:** Rasch summary statistics of the Japanese version of the Visual Discomfort Scale for 428 persons.

	Mean	*SD*	Min	Max
Total score	12.59	9.63	0	57
Location (standard error)	-1.99 (0.44)	1.25 (0.28)	-5.66 (0.25)	1.69 (1.83)
**Real:** *RMSE* = 0.54, adjusted *SD* = 1.13, separation = 2.10, person reliability = 0.82				
**Model-based:** *RMSE* = 0.52, adjusted *SD* = 1.14, separation = 2.18, person reliability = 0.83				
Standard error of person mean = 0.06, Correlation between person raw scores and measures = 0.91				
Cronbach alpha (standard error of measurement) = 0.90 (3.01)				

*SD*: standard deviation; *RMSE*: root mean square error

**Table 4:** Descriptive statistics of the measures in Survey 1 and 2.

		Mean	*SD*	Min	Max	Skew	Kurtosis	Cronbach’s *α*
Survey 1 (*N* = 428)								
	VDS	12.59	9.63	0	57	1.27	2.23	0.90
Survey 2 (*N* = 118)								
	VDS (Time 1)	9.53	10.13	0	69	2.53	9.89	0.95
	VDS (Time 2)	8.60	9.06	0	57	1.98	6.13	0.94

*N*: number; *SD*: standard deviation; VDS: Visual Discomfort Scale.

We obtained a person reliability of 0.82 (Table 3) and an item reliability of 0.98 (Table 2). These measures suggest that the Japanese VDS is sufficient for classifying respondents and verifying item hierarchy, according to the previously explained criteria in which a person reliability larger than 0.80 and item reliability larger than 0.90 are desirable [62]. Moreover, the Cronbach’s alpha was sufficiently high (0.90, Table 3), again suggesting good internal consistency. The targeting of 1.99 was between 1.00 and 2.00, and suggested that the Japanese VDS has a medium level of matching between item difficulty and respondents’ ability [65]. Several items showed “mild” DIF for sex (i.e., differential of item measures between 0.50 and 1.00 [65]); 0.67 for the item 4 and 0.54 for the item 5, while the other items showed ignorable DIFs, lower than 0.34. There were no substantial DIFs for age, suggested by all items showed the differential lower than 0.45. Finally, some items showed substantial DIFs for the four headache categories; item 1: *χ*^2^(3) = 7.95, *p* = 0.047; item 4: *χ*^2^(3) = 33.64, *p* < 0.001; item 5: *χ*^2^(3) = 12.10, *p* = 0.007; item 6: *χ*^2^(3) = 10.44, *p* = 0.02; item 8: *χ*^2^(3) = 14.39, *p* = 0.002. The other items did not show DIFs (*χ*^2^s < 6.18, *p*s > 0.10). In sum, since several items showed mild or substantial DIF for sex and/or headache, the Japanese VDS exhibited medium quality in terms of DIF [65].

Items 7 and 19 might be considered as candidates to be removed from the VDS because they showed the fit statistics that indicated a misfit to the model. We analyzed the 21-item version of the Japanese VDS without items 7 and 19 using the same strategy explained above (see S5 File for detailed results). Even after the removal of two items, item 1 still showed an outfit mean-square of 1.34, thus exceeding the criterion range [65]. We further analyzed the 20-item version without items 1, 7, and 19. The infit and outfit mean-squares for the 20-item version were within the criterion range [65], suggesting a good fit with the model. PCA revealed that the 20-item version showed dimensionality comparable to the 23-item version. The 20-item version also showed reliability and DIF comparable to the 23-item version; however, person reliability of 0.78 did not reach the criterion of 0.80 [62, 63]. Low person reliability indicates that the scale may need more items [62]. Moreover, the 20-item version showed a low level of targeting of 2.21, larger than 2.00 [65]. In sum, even when we removed three items that seemed to misfit the model, some aspects of psychometric property (e.g., dimensionality and DIF) were comparable with its full version. However, importantly, the 20-item version would be inferior in person reliability and targeting to the full version. Therefore, we decided to utilize the 23-item full version of the Japanese VDS in the subsequent analyses.

#### Known-groups validity with migraine

**Fig 3:**
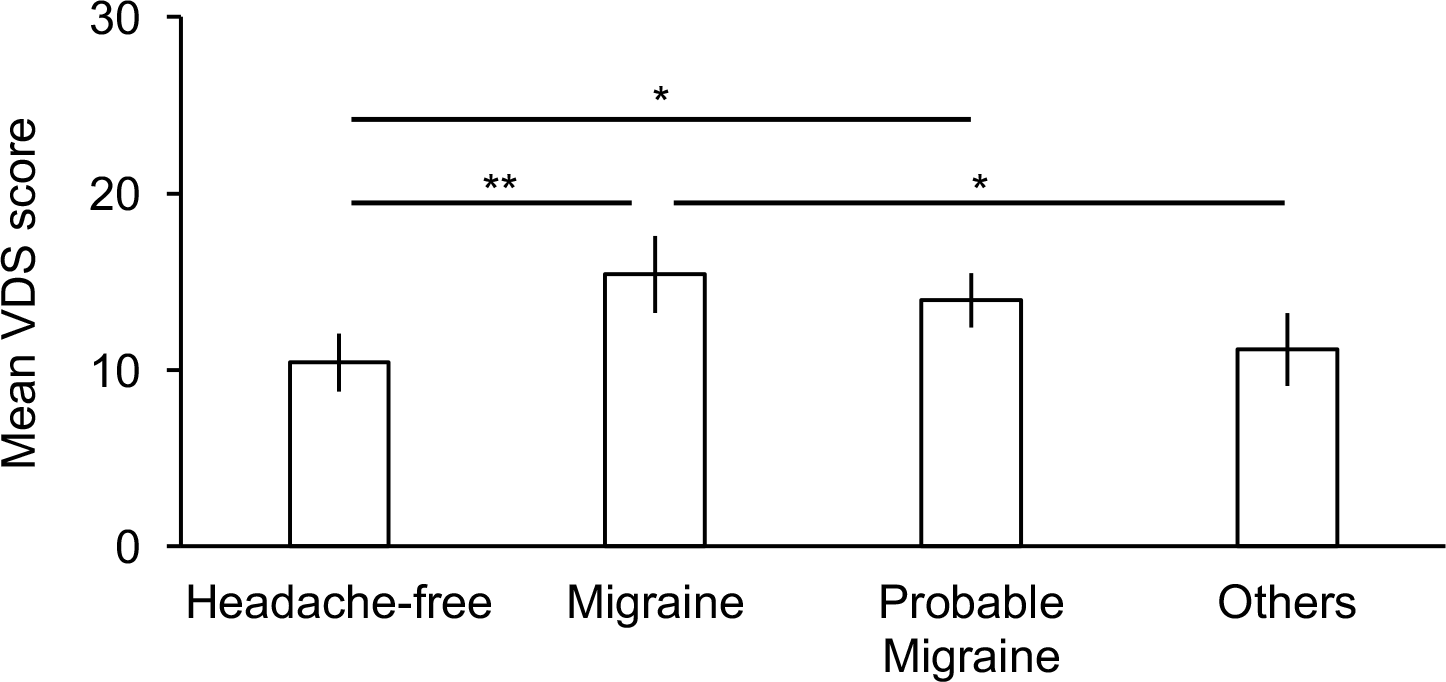
Visual Discomfort Scale scores in participants with and without migraine, probable migraine, or other headaches. Error bars denote 95% confidence interval. Asterisks denote significant differences (*p < 0.05, **p < 0.01, Bonferroni-corrected). VDS: Visual Discomfort Scale.

The VDS score did not correlate with age (*r*(426) = -0.06, *p* = 0.20), but was higher in female than in male participants (*Mean*_male_ = 10.73, *SD*_male_ = 8.92; *Mean*_female_ = 13.95, *SD*_female_ = 9.91; *t*(426) = 3.46, *p* = 0.001, *d* = 0.34), which is in line with the results of a previous study [23]. Moreover, age did not differ among the four headache categories (*F*(3,424) = 1.48, *p* = 0.22, *η*^2^_p_ = 0.01), while the distribution of the four headache categories differed between the sexes (*χ*^2^(3) = 40.00, *p* < 0.001, Cramer’s *V* = 0.31; see also S3 File). These findings suggested that female participants were more likely to have migraine or probable migraine (24.29 and 38.87%, respectively) than male participants were (7.73 and 26.52%), which is consistent with previous epidemiological evidence [69, 70]. Thus, we performed an ANCOVA on the VDS score with the headache categories as a between factor and sex as a covariate. At first, we confirmed that the ANCOVA satisfied the assumption of the parallelism of regression lines (*F*(3,420) = 0.85, *p* = 0.47, *η*^2^_p_ = 0.006) and the significance of regression by the covariate (*t* = 2.09, *p* = 0.04). As shown in Fig 3, the ANCOVA revealed a significant effect of headache categories on the VDS score (*F*(3,423) = 5.64, *p* = 0.001, *η*^2^_p_ = 0.04). A post-hoc test revealed that the Migraine participants (*Mean* = 15.86, *SD* = 9.99) scored significantly higher than the Headache-free (*Mean* = 10.06, *SD* = 8.22; *p* = 0.003, Bonferroni-corrected) and Others participants did (*Mean* = 10.94, *SD* = 8.32; *p* = 0.04). Moreover, the Probable Migraine participants (*Mean* = 14.11, *SD* = 10.56) also scored higher than the Headache-free participants did (*p* = 0.02), but did not differ from the Migraine (*p* = 0.99) and Others (*p* = 0.21) participants. These results suggest the known-groups validity of the Japanese VDS, which can discriminate the individuals with migraine, who are more likely to experience visual discomfort than headache-free individuals are [3, 15–21], from the individuals with other primary headaches and without any primary headaches who also experience visual discomfort from various visual stimuli [9–12]. Consequently, the higher VDS score in the Migraine participants than that in the Probable Migraine participants might also be expected. However, this was not the case for our results, perhaps because probable migraine was similar to full-blown migraine in terms of headache characteristics and accompanying symptoms [48, 49], and might be accompanied by reports of visual discomfort comparable to those accompanying full-blown migraine, or simply because there may be a limitation in the known-groups validity of the Japanese VDS.

## Survey 2: Test-retest reliability

The primary aim of this online survey recruiting Japanese adults was to examine the stability of the Japanese VDS in terms of test-retest reliability. Secondarily, the internal consistency of the Japanese VDS was examined again.

### Materials and methods

#### Participants and procedures

A total of 227 Japanese adults were recruited from Yahoo Crowdsourcing, which is an online labor market similar to Amazon Mechanical Turk [71]. They participated via SurveyMonkey (http://www.surveymonkey.com), using their own computer. At this Time 1, participants were first given the ethical statement and consent form. Participants, who provided informed consent, provided their sex and age, completed the Japanese VDS, and reported their email address. Finally, participants were thanked and given a cash voucher equivalent to 100 Japanese yen (approximately 0.9 US dollars). Due to the repeated participation, 24 participants, who showed an identical IP address, sex, and age, were excluded in order to ensure a level of data quality by screening invalid respondents [72], although previous studies have suggested that data collected from online samples can be psychometrically reliable and valid, and comparable with data collected from students and community samples [73–77] even when using Japanese online labor markets [78].

Two weeks after Time 1, we sent an email asking the remaining 203 participants to participate in the retest of the VDS (Time 2) via SurveyMonkey. A total of 127 of the participants consented to participate, and again reported their sex and age, completed the Japanese VDS, and were asked to report the email address, similar to that done at Time 1. Participants were given a cash voucher equivalent to 100 Japanese yen. In order to screen invalid respondents, we excluded nine participants who reported inconsistent sex, age, and email address at Time 1 and 2 (note that age increment of one year was acceptable). Finally, we analyzed the data from 118 participants (38 females; mean age = 40.20 years, *SD* = 8.42 years, range = 22 to 73 years).

#### Measure

Participants completed the Japanese VDS at Time 1 and 2.

#### Data analysis

**Fig 4:**
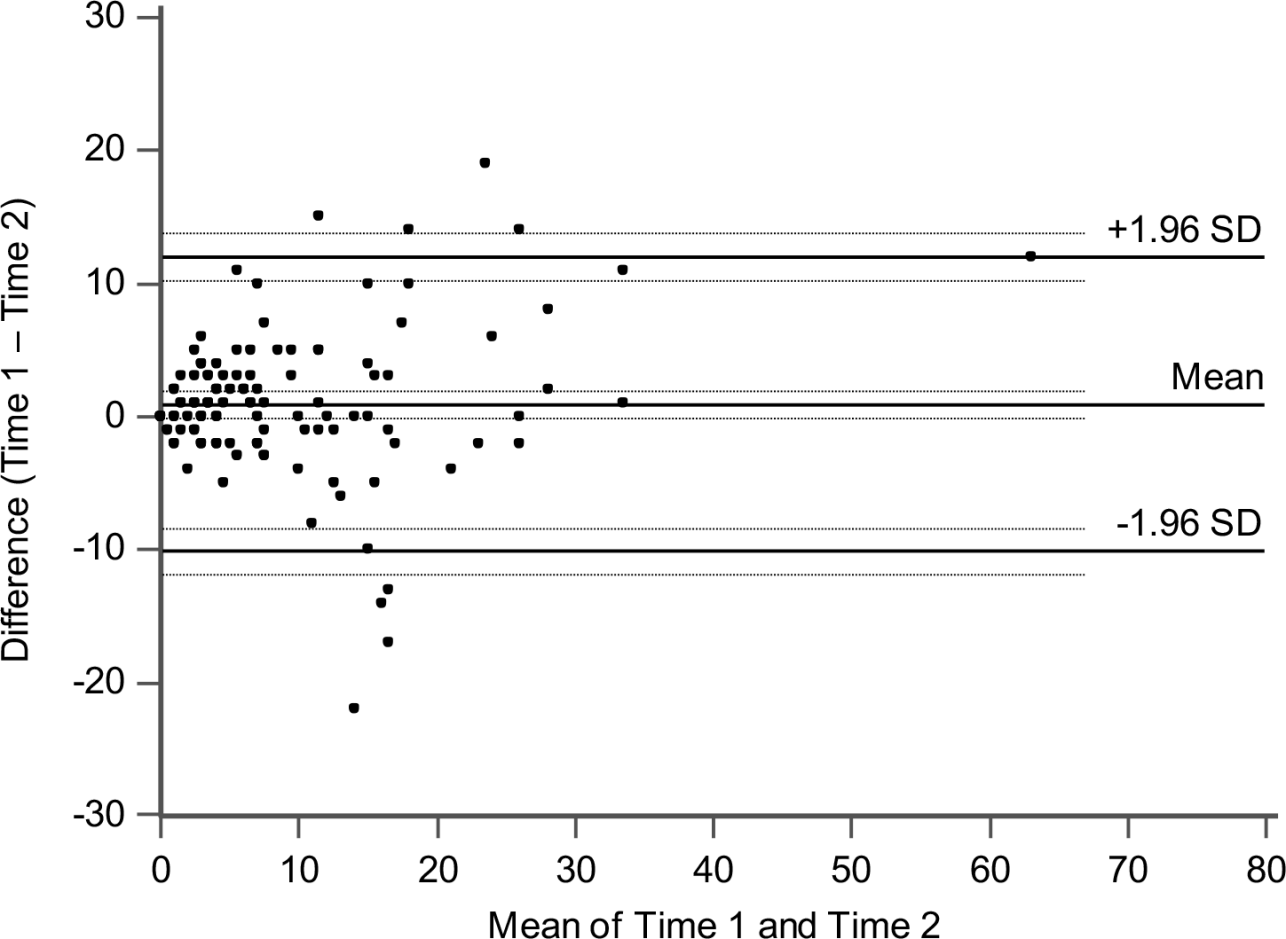
Bland-Altman plot for the Visual Discomfort Scale scores in Survey 2. Each dot represents data from a single participant. Solid lines indicate the averaged difference and the upper and lower limits of agreement. Broken lines indicate 95% confidence intervals.

Descriptive statistics including internal consistency (Cronbach’s alpha) were reported. To examine the test-retest reliability of the VDS, correlation analysis between the VDS scores at Time 1 and 2, and a paired two-tailed *t*-test comparing Time 1 and 2, were performed. The Pearson’s correlation coefficient (*r*) was used if the data normally distributed based on the Shapiro-Wilk’s test, while the Spearman’s correlation coefficient (*ρ*) was used if the data was not normally distributed. The Welch’s *t*-test with corrected degrees of freedom was used when the data violated the assumption of homogeneous variance in accordance with the Levene’s test. We further used the Bland-Altman method [79] to quantify the agreement between Time 1 and 2 by calculating the limits of agreement (LoA) that was estimated by mean ± 1.96 *SD* of differentials between the two measurements from the same individuals. We also reported the 95% confidence interval for the mean differential and the upper and lower limits according to a recent guideline [80]. Our sample size of 118 was above 100, which has been recommended for the Bland-Altman method [80]. The Bland-Altman plot (Fig 4) was generated using MedCalc 17.6 (MedCalc Software, Ostend, Belgium). Moreover, we examined any effect of sex and age on the VDS score at Time 1 and 2, similar to that in Survey 1. If an effect was observed, we performed the post-hoc correlation analysis partialling out the effect of sex and/or age.

### Results and discussion

Descriptive statistics have been reported in Table 4. We confirmed that age did not correlate with the VDS scores at Time 1 (*ρ*(116) = 0.07, *p* = 0.44) and Time 2 (*ρ*(116) = 0.12, *p* = 0.20), and there was no difference in age between sexes (*t*(116) = 0.74, *p* = 0.46, *d* = 0.15). Moreover, there were no sex differences in the VDS scores at Time 1 (*t*(116) = 0.39, *p* = 0.70, *d* = 0.08) and Time 2 (*t*(98.22) = 0.76, *p* = 0.45, *d* = 0.13). Importantly, as for test-retest reliability, we found that the VDS scores at Time 1 and 2 were strongly and positively correlated (*ρ*(116) = 0.79, *p* < 0.001), but did not differ significantly (*t*(117) = 1.79, *p* = 0.08, *d* = 0.10). As shown in Fig 4, the Bland-Altman method revealed a mean difference of 0.93 (95% confidence interval: -0.10 to 1.96) between the VDS scores at Time 1 and Time 2 and the LoA ranged from -10.13 to 12.00 (95% confidence interval of lower limit: -11.90 to -8.37; upper limit: 10.23 to 13.76). This suggests that, in comparison with a previously reported LoA ranging from -18.44 to 17.92 by using the original VDS [24], the Japanese VDS showed narrower LoA, indicating a comparable or even better reproducibility of measurement of the Japanese version. Finally, both the VDS at Time 1 and 2 showed high internal consistency with alphas of 0.95 and 0.94, respectively. In sum, these results suggested good stability (i.e., test-retest reliability) of the Japanese VDS and again confirmed its sufficient internal consistency.

The VDS scores at Time 1 and 2 in Survey 2 seemed lower than those in Survey 1 (Table 4). Indeed, a follow-up analysis using a two-tailed unpaired *t*-test indicated that this was true (Survey 1 versus Time 1 in Survey 2: *t*(544) = 3.02, *p* = 0.006, *d* = 0.31; Survey 1 versus Time 2 in Survey 2: *t*(544) = 4.04, *p* = 0.002, *d* = 0.42; Bonferroni-corrected). This might be due to a sampling bias whereby Survey 1 (247 in 428; 57.71%) included a higher proportion of female participants than Survey 2 did (38 in 118; 32.20%), *χ*^2^(1) = 25.74, *p* < 0.001, *φ* = 0.22. Females are more likely to have migraine [69, 70] which can be accompanied by heightened visual discomfort [3, 15–22]. Thus, the Survey 1, in which the majority of the participants were female, might have resulted in higher VDS scores. However, we should be cautious about this speculation based on cross-survey comparison because Survey 2 did not administer the migraine screening questionnaire.

## General discussion

In the current study, the Japanese version of the VDS was developed for assessing the everyday experiences of visual discomfort that includes distorted perception, somatic symptom, and uncomfortable feeling towards visual stimuli.

A Rasch modeling analysis revealed that almost all items of the Japanese VDS well fitted to a unidimensional structure, although the degree of fitting was low relative to that exhibited by the original version [22, 23] in terms of model-fit statistics and dimensionality. First, the Rasch fit statistics, i.e., averaged infit mean-square of 1.03 (0.73–1.31) and outfit mean-square of 0.96 (0.71–1.42), can be acceptable when based on an established lenient criterion (0.50–1.50) [62]. For instance, the original authors reported that the averaged infit and outfit mean-square were 0.97 and 0.92, respectively (range unavailable) [22], and other researchers reported that the averaged infit mean-square was 1.05 (range 0.72–1.34) and outfit mean-square was 0.94 (range 0.70–1.35) [23]. These results suggest that the VDS may have a Rasch-based unidimensional structure providing a non-language-specific measurement. However, we analyzed the data using a more stringent criterion (0.70–1.30) [63, 65], and found that items 1, 7, and 19 showed the mean-square statistics violating the criterion. We again examined the Rasch-based psychometric properties of the scale without these three items and found comparable qualities of the full and reduced versions of the Japanese VDS except for better person reliability and targeting in the full version. In addition, the current study, as a translation study of an established scale, should be careful about item reduction to afford future comparative and/or meta-analytic studies. Thus, we decided to retain all the items in the Japanese version of the VDS.

Second, the Rasch-based PCA revealed that only 38.1% of raw variance was explained by measures. This value was below an established criterion [65] and the value of 73.5% reported by a previous study using a college student sample completing the original VDS [23]. These findings suggest that the Japanese version might have a low degree of unidimensionality. Our PCA also revealed latent second and third dimensions (i.e., first and second PCA contrasts). The first contrast remarkably loaded on items 6, 7, 8, and 23 (Table 1). These items seemed to pertain to re-reading and slowing down of reading speed due to visual discomfort. Similarly, in the original version, these items included in the “Re-reading” component potentially underlying the VDS [42]. On the other hand, the second contrast loaded on items 1 to 5 (Table 1). These items seemed to pertain to tearing, drying, or straining of the eyes, and headache triggered by visual stimuli such as striped patterns, printed texts, and lights. Again, in the original version, these items comprised the “Headache/soreness” factor extracted by a PCA performed on the residuals [23, 42]. Although it has been suggested that there are various sources and aspects of visual discomfort, which can be classified into externally triggered symptoms (e.g., tearing, dryness of the eyes) and internally triggered symptoms (e.g., headache and strain) [81], it is evident that the Japanese version’s items that loaded in the second contrast included both external and internal triggering symptoms (i.e., items 1 to 3 pertained to external and internal symptoms, and items 4 and 5 only pertained to internal symptom). In this sense, our results may suggest that the Japanese VDS can measure visual discomfort as a single construct, although several aspects might underlie the single construct [81]. However, most importantly, highly strong correlations between item clusters, divided by each of first and second PCA contrasts, suggested that all items can measure virtually the same construct, namely, they exhibited unidimensionality. Taken together, these results also suggested that the Japanese VDS may show not only unidimensionality but also noticeable unexplained variances that simply represent random noise. Given the large difference in the amount of explained variance between the original [23] and Japanese version of the VDS, further elaboration of the Japanese version may be needed.

The scale score of the Japanese VDS (mean ± *SD*: 12.59 ± 9.63 for Survey 1; 9.53 ± 10.13 for Time 1 in Survey 2) can be considered as comparable to that of the original version. The first development study in Australia (*N* = 514) reported a mean scale score of 11.88 (*SD* unavailable) [22], while a following cohort study in the United States reported a mean score of 15.40 ± 10.20 (*N* = 571) [23]. However, higher VDS scores have been reported by other studies with smaller samples. For example, 23 volunteers in the United States showed a VDS score of 22.00 ± 14.70 [24], and 68 other volunteers scored 21.20 ± 13.10 [43]. This variation suggests that the VDS might be prone to sampling biases, such as prevalence of migraine, which is likely to increase visual discomfort [3, 15–22] and ratio of female participants, who are more likely to have migraine [69, 70]. Indeed, these biases were also suggested in our data from Survey 1 and 2. Thus, researchers should be careful about these methodological concerns when using the VDS, although they can still obtain benefits from the fact that the original and Japanese versions provided comparable scale scores.

### Reliability

We found a sufficient level of Rasch person reliability (0.82) in the Japanese VDS, which was comparable to 0.87 reported for the original version [23]. This suggests that the Japanese VDS has the capacity to reproducibly distinguish individuals with high or low proneness to visual discomfort. On the other hand, the Rasch item reliability, which indicates the reproducibility of the items’ hierarchy and/or given scores for each item, and which has not been previously reported in other studies, was also sufficiently high in the present study (0.98). In line with internal consistency of 0.91 for the original version [22], we also reported sufficiently high alphas of the Japanese VDS, ranging from 0.90 to 0.95 (Table 4) as an index of internal consistency (see [82] for controversy over the validity of Cronbach’s alpha).

In the present study, we used several indices to verifying the stability (i.e., test-retest reliability) of the Japanese VDS. There was strong positive correlation between the Japanese VDS scores at Time 1 and 2 of Survey 2. In line with this, only Borsting et al. [24], to our knowledge, have reported good stability of the original VDS. They reported a sufficient intraclass correlation coefficient between two sessions with an interval of approximately thirteen months, as well as the averaged differential between these sessions with the LoA, which was indeed wider than that of the Japanese VDS, suggesting a better reproducibility of the Japanese version. Taken together, the good stability of the Japanese and original versions does not only indicate their ensured psychometric quality but can also reflect that visual discomfort might be chronic because it may stem from perceptual and/or neuronal traits [1, 2, 4].

### Validity

The validity of the Japanese VDS was supported by ensuring the known-groups validity with the migraine headache morbidity in Survey 1.

There is consensus among researchers that increased visual discomfort is observed more frequently in individuals with migraine than in those without primary headaches, in terms not only of the VDS score [22, 26, 46, 47, 54], but also of behavioral responses [3, 15–17, 20, 83] and neural activities in visual cortices [18, 84]. Previous studies (Table 5) have consistently reported that individuals with migraine, regardless of comorbidity of visual aura, score higher on the VDS than those without primary headaches do [26, 46, 47, 54]. In line with this, our results from Survey 1 revealed that participants with migraine scored higher on the Japanese VDS than those with other types of primary headaches and those without primary headaches did. This suggests the known-groups validity of the Japanese VDS, although the difference between the participants with potential visual aura and the headache-free participants did not reach significance perhaps due to the shortage or imbalance of sample sizes. However, the Migraine and Probable Migraine participants did not show a difference in the VDS score, suggesting that the Japanese VDS may be limited in terms of distinguishing between individuals with full-blown migraine and those with other primary headaches and without any primary headaches. On the other hand, we should point out that the VDS score observed in Survey 1 was higher than that at Time 1 and 2 in Survey 2. This may be because Survey 1 included a higher proportion of females, who are more likely to have migraine than males [69, 70]. To speculate, this cross-survey difference in the VDS score might reflect the different proportion of the individuals with migraine and may also suggest the known-groups validity of the Japanese VDS.

**Table 5:** Differences in Visual Discomfort Scale scores between individuals with migraine and without primary headaches reported by the previous and current studies.

	Migraine group	Headache-free group	Test for group difference
Conlon et al. [22]	Without reading difficulty: mean = 10.49 (1.0), *N* = 69. With occasional reading difficulty: mean = 19.27 (1.2), *N* = 34. With regular reading difficulty: mean = 30.91 (0.5), *N* = 23.	Without reading difficulty: mean = 6.35 (1.2), *N* = 252. With occasional reading difficulty: mean = 15.36 (1.0), *N* = 105. With regular reading difficulty: mean = 25.90 (1.0), *N* = 31.	**Main effect of headache**: *F*(1, 508) = 17.52, *p* < 0.05. **Main effect of reading difficulty**: *F*(2, 508) = 127.9, *p* < 0.05. No interaction between headache and reading difficulty (*F* and *p* values unavailable). Post-hoc comparisons unavailable.
Shepherd et al. [46, 47]	With visual aura: mean = 25.4 (13.6), *N* = 14. Without visual aura: mean = 22.1 (13.6), *N* = 14. Total: mean = 23.8 (13.5), *N* = 28.	Mean = 11.6 (8.2), *N* = 14.	**With visual aura versus Headache-free**: *t*(26) = 3.2, *p* = 0.003. **Without visual aura versus Headache-free**: *t*(26) = 2.5, *p* = 0.02. No difference between with and without visual aura: *t*(26) = 0.6, *p* = 0.50.
Datta et al. [26]	With visual aura: median = 9 (4–16), *N* = 25. Without visual aura: median = 8 (5–16), *N* = 17.	Median = 4 (1–7), *N* = 19.	**With visual aura versus Headache-free**: *p* = 0.008. **Without visual aura versus Headache-free**: *p* = 0.005. No difference between with and without visual aura: *p* = 0.93. *U* statistics unavailable.
Cucchiara et al. [54]	With visual aura: median = 9 (5–16), *N* = 51. Without visual aura: median = 8 (6–14), *N* = 45.	Median = 3 (1–6), *N* = 45.	**With visual aura versus Headache-free**: *p* < 0.0001. **Without visual aura versus Headache-free**: *p* < 0.0001. No difference between with and without visual aura: *p* = 0.70. *U* statistics unavailable.
Current study	With visual aura: mean = 16.92 (13.39), median = 11.00 (5.25–30.50), *N* = 12. Without visual aura: mean = 15.66 (9.32), median = 14.50 (8.75–21.25), *N* = 62. Total: mean = 15.86 (9.99), median = 14.00 (8.00–22.00), *N* = 74.	Mean = 10.06 (8.22), median = 8.00 (4.00–14.25), *N* = 130.	With visual aura versus Headache-free: *t*(11.78) = 1.74, *p* = 0.11. **Without visual aura versus Headache-free**: *t*(190) = 4.23, *p* < 0.001. **Migraine (with and without visual aura) versus Headache-free**: *t*(202) = 4.48, *p* < 0.001. No difference between with and without visual aura: *t*(13.14) = 0.31, *p* = 0.76.

*N*: number. Values in parentheses following mean and median indicate standard deviation and interquartile range, respectively. Statistically significant group differences are indicated in bold. All of cited previous studies used the original version of the Visual Discomfort Scale [22].

### Limitations

The current study has several limitations and potential biases [85]. First, there was a potential shortcoming in the translation process. Formal pretest after confirmation of the consistency between the original and back-translated versions was recommended in order to ensure appropriate wording and comprehension of the developed items [86]. However, in our translating process, only two Japanese individuals reviewed the Japanese VDS before the main surveys. Thus, besides the issue of reliability of this review, an obsequiousness bias might have ignored flaws in the wording and comprehension, deteriorating the psychometric quality of the Japanese VDS. However, as they were naïve to our study purpose and not our acquaintances, the obsequiousness bias would be unlikely. Although the present results suggested the reliability and validity of the Japanese VDS, the use of more rigorous methods in future studies may help improve the Japanese VDS.

Second, the migraine screening questionnaire have not been formally validated. However, previous behavioral evidence has partially supported the convergent and known-groups validities of this measure (see Measures in Survey 1 for details). Consequently, we decided to adopt it as an appropriate measure to assess the validity of the Japanese VDS. Although our results suggested the validity of the Japanese VDS, these results were possibly affected by an instrument bias due to the quasi-valid migraine screening questionnaire. Thus, further psychometric evidence supporting the validity and reliability of these measures would be beneficial for future investigations of visual discomfort and its related conditions (e.g., migraine).

Third, there were substantial differences in the samples and sampling methods between our surveys. One might argue that comparable samples are needed to assess the reliability and validity of a developed scale in separate studies. Indeed, our results might have been affected by potential membership biases where samples were collected from colleges (Survey 1) and online labor markets (Survey 2). Specifically, the younger sample with a higher proportion of females in Survey 1 resulted in higher VDS scores than in Survey 2. Moreover, online sampling might induce an attention bias because the examiner cannot directly see the participants’ behavior. It has been suggested that online sampling has potential issues with responding behaviors such as inattentiveness and cheating for factual questions [87]. However, Survey 2 resulted in better internal consistency of the Japanese VDS than in Survey 1. In addition, several studies have successfully examined test-retest reliability of measures of personality and psychiatric traits by using online samples [71, 76, 88–90]. Nevertheless, future replication studies, especially on the test-retest reliability, using comparable samples collected by paper-and-pencil methods would be beneficial to further support the reliability of the Japanese VDS.

Fourth, while the coincidence of scoring high on the VDS and having migraine in Survey 1 was theoretically expected [22, 26, 46, 47, 54] and indeed supported the known-groups validity of the Japanese VDS, we cannot rule out potential response biases inherent in self-report methods, which may have affected our data. For example, there might have been a recall bias, where those with migraine, who may have vivid and frequent recall of their headache and visual discomfort, tend to agree more with questions relating to headache and visual discomfort. Although only items 4 and 5 of the VDS directly ask about headache triggered by visual stimuli, the recall bias may have potentially influenced responses to all items of the VDS. However, there may also have been an obsequiousness bias where participants, who were aware of their presence or absence of headache, attempted to agree or disagree more with the VDS items. If this was the case, the differences between the four headache categories we found have been in fact exaggerated. Thus, future studies should further evaluate the construct validity of the Japanese VDS by using control measures, which are expected not to correlate with actual conditions of visual discomfort and/or migraine, and by conducting objective measurements of, for instance, the unpleasantness and distorted perceptions induced by visual stimuli (e.g., repetitive stripes, texts) in individuals with various degrees of VDS scores and in those with and without migraine, as previously done in the study in which the original VDS was developed [22].

Finally, although the VDS, by definition, measures visual discomfort induced by two-dimensional visual properties in viewing static visual stimuli and reading texts, visual discomfort related to stereoscopic vision may also be partially measured by the VDS, of which some items (e.g., items 11 and 12) ask about blur and diplopia when reading [23] induced by accommodation and vergence [91]. This might serve as a potential instrument bias underlying our findings on the relationship between visual discomfort and migraine. However, to our knowledge, no studies have reported abnormal stereoscopic function in individuals with migraine. Thus, to speculate, it is unlikely that the potential confounding with the stereoscopic visual discomfort affected our findings. Given another line of research has established detailed assessments of the stereoscopic visual discomfort [92], future studies should acknowledge the different assessments for visual discomfort stemming from different origins [81], and aim to provide an integrated understanding of visual discomfort.

## Conclusions

The Japanese version of the VDS has a unidimensional structure fitted to the Rasch model, which is in line with the original version [22], although researchers should be careful while using the Japanese version, because its psychometric properties can be accompanied by a noticeable amount of unexplained variances attributable to random noise rather than to another dimension. The Rasch reliability, internal consistency, and test-retest reliability of the Japanese VDS were sufficient. The construct validity was supported by the results indicating that individuals with migraine, who were prone to experiencing visual discomfort, indeed scored high on the Japanese VDS. To our knowledge, this is the first study to validate the translated VDS. We hope that the Japanese and the upcoming other-language translated versions will contribute to experimental and epidemiological studies for deeper understanding of the mechanisms underlying visual discomfort and its inter-individual variability.

## Supporting information

S1 File Japanese version of the Visual Discomfort Scale. (DOCX)

S2 File Migraine screening questionnaire. An English version of the questionnaire originally written in Japanese.
(DOCX)

S3 File Summary of headache characteristics and prevalence in Survey 1. (DOCX)

S4 File Rasch statistics of the Japanese version of the Visual Discomfort Scale for 418 persons without minimum extreme. (DOCX)

S5 File Rasch-based psychometric properties of the 20-item version of the Japanese Visual Discomfort Scale. (DOCX)

S6 File Raw data from Survey 1 and 2. (XLSX)
