# Supplementary material for: Development of the Japanese version of the Visual Discomfort Scale
Source: PLoS One. 2018 Jan 11;13(1):e0191094. doi: 10.1371/journal.pone.0191094 (PMC5764345; doi:10.1371/journal.pone.0191094)
Supplement: S1 File — (DOCX) [file pone.0191094.s001.docx]

各質問について，最もよくあてはまる選択肢の欄に◯を記入してください。

|  |  | そういった経験はない | 時々ある（年に数回） | たびたびある（２〜３週に１回） | ほぼ毎日ある |
| --- | --- | --- | --- | --- | --- |
| 1. | 縞模様を見ているとき，「涙目になる」「目が充血する」「目が痛む」「目がつらい」「目が疲れる」「目が乾く」「目がゴロゴロする」「頻繁に目をこする」といった問題が起こることはありますか？ |  |  |  |  |
| 2. | くっきりと印刷された新聞や雑誌をしばらくの間読んだ後で，「涙目になる」「目が充血する」「目が痛む」「目がつらい」「目が疲れる」「目が乾く」「目がゴロゴロする」といった問題が起こることはありますか？ |  |  |  |  |
| 3. | 蛍光灯の光で仕事をしているとき，「涙目になる」「目が充血する」「目が痛む」「目がつらい」「目が疲れる」「目が乾く」「目がゴロゴロする」といった問題が起こることはありますか？ |  |  |  |  |
| 4. | 蛍光灯の光で仕事をしていて，頭が痛くなることはどの程度ありますか？ |  |  |  |  |
| 5. | くっきりと印刷された新聞や雑誌を読んでいて，頭が痛くなることはありますか？ |  |  |  |  |
| 6. | 何かを読んでいて，同じ行にある同じ単語を２回読んでしまうことはありますか？ |  |  |  |  |
| 7. | 小説や雑誌を読んでいるとき，鉛筆や指で読んでいる箇所をたどらないと，どこを読んでいるのかわからなくなってしまうことはありますか？ |  |  |  |  |
| 8. | 何かを読んでいて，同じ行をうっかり２回読んでしまうことがありますか？ |  |  |  |  |
| 9. | くっきりと印刷されたものを読んでいるとき，文字がぼやけたり焦点が外れたりするのを防ぐために，目を細めないといけないことはありますか？ |  |  |  |  |
| 10. | くっきりと印刷されている文章を読んでいるとき，文字がいったん遠くに薄れていき，再び現れるような感じに陥ることはありますか？ |  |  |  |  |
| 11. | くっきりと印刷されている文章を読んでいるとき，文字がぼやけることはありますか？ |  |  |  |  |

各質問について，最もよくあてはまる選択肢の欄に◯を記入してください。

|  |  | そういった経験はない | 時々ある（年に数回） | たびたびある（２〜３週に１回） | ほぼ毎日ある |
| --- | --- | --- | --- | --- | --- |
| 12. | 何かを読んでいるとき，文字が二重に見えることはありますか？ |  |  |  |  |
| 13. | 何かを読んでいるとき，ページの単語が動いているように見えたり，ページから浮かび上がって見えたりすることがありますか？ |  |  |  |  |
| 14. | くっきりと印刷されている文書を読んでいるとき，目で文字を追うのが困難に感じることはありますか？ |  |  |  |  |
| 15. | 白地に黒い文字が印刷してあるページを読んでいるとき，白い背景が文字を侵食してしまい，文字が読みづらくなることはありますか？ |  |  |  |  |
| 16. | 白地に黒い文字が印刷してあるページを読んでいるとき，白い背景からの光の反射が気になり，ページを絶えず動かしたり，何度も瞬きをしたりしなければいけないことがありますか？ |  |  |  |  |
| 17. | １行の中で１つあるいは２つ以上の単語に焦点をあわせるのが困難になることはありますか？ |  |  |  |  |
| 18. | 何かを読んでいるとき，ページ上の文字がちらついたり揺らめいたりするように見え，読むのが困難になることはありますか？ |  |  |  |  |
| 19. | 蛍光灯の光や直射日光の下で何かを読んでいるとき，ページの白い背景が反射することで，ページを絶えず動かしていないと文字がはっきりとは見えなくなることがありますか？ |  |  |  |  |
| 20. | 何かを読んでいるとき，ページ上で絶えず視線を移動させる，あるいは頻繁に瞬きをする，目をこするなどしないと文字が見づらいですか？ |  |  |  |  |
| 21. | 文字の下の白い背景が動く，ちらつく，揺らめくように見えて，文字が読みづらいことがありますか？ |  |  |  |  |
| 22. | 何かを読んでいるとき，単語や文字が広がるように見えることはありますか？ |  |  |  |  |
| 23. | １から２２のいずれかの問題のために，何かを読むのが遅くなっていると感じますか？ |  |  |  |  |
