# Supplementary material for: Development of the Japanese version of the Visual Discomfort Scale
Source: PLoS One. 2018 Jan 11;13(1):e0191094. doi: 10.1371/journal.pone.0191094 (PMC5764345; doi:10.1371/journal.pone.0191094)
Supplement: S2 File — An English version of the questionnaire originally written in Japanese. (DOCX) [file pone.0191094.s002.docx]

1. Have you ever experienced a headache except those attributable to a common cold or hangover? Please circle “Yes” or “No.”

- Yes
- No

**If you answered “Yes,” please proceed to the following questions.**

**If you answered “No,” please stop answering.**

**You will be asked questions about your headaches. Please circle the choice most applicable to you.**

1. When did your headaches begin to occur?

- More than a year ago (from the age of years)
- A few months ago
- A few weeks ago
- A few days ago

1. How many headaches have you ever had?

- Once
- Two to four times
- Five times or more

1. Is the type of headache always the same or are there several types?

- Always the same type
- More than two types

1. How often do you have headaches?

- Several times a year
- About ten times a year
- One to three times a week
- Almost every day
- Every day during a certain period, but none during other periods

1. How long do your headaches last unless you take medications?

- Less than four hours
- Four hours to three days
- Three days or more
- Almost every day

**You will be asked questions about the characteristics of your headaches.**

1. Which area hurts mainly? Please circle the choices applicable to you.

- One side
- Both sides
- Occipital area and neck
- Top of head
- Whole head
- Behind either eye

1. What kind of pain do you experience during your headaches? Please circle the choices applicable to you.

- Pulsating
- Tightening and squeezing
- Stabbing
- Sharp and instantaneous

1. How intense is the pain of your headaches if you do not take medications? Please circle the choice most applicable to you.

- Severe pain completely preventing you from normal activities (i.e., severe pain)
- Pain severe enough to interfere with your normal activities (i.e., moderate pain)
- Pain not restricting your normal activities (i.e., mild pain)

1. Do you experience your headaches worsened by daily physical activities (e.g., walking, climbing stairs) and causing avoidance of such activities? Please circle “Yes” or “No.”

- Yes
- No

**You will be asked questions about symptoms accompanying your headaches. Please circle “Yes” or “No.”**

1. A zigzag figure near the fixation point presents and part of the visual field is blurred and vanished before headaches.

- Yes
- No

1. Headaches accompanied by nausea and vomiting.

- Yes
- No

1. Become sensitive to light and sound during headaches.

- Yes
- No

1. Become sensitive to odor during headaches.

- Yes
- No

1. Experience stiffness or pain of the neck or shoulder before headaches.

- Yes
- No

1. The eye on the side of headache is congested and tears during headaches.

- Yes
- No

1. The eyelid on the side of headache closes during headaches.

- Yes
- No

1. Nasal congestion during headaches.

- Yes
- No
