# Supplementary material for: Development of the Japanese version of the Visual Discomfort Scale
Source: PLoS One. 2018 Jan 11;13(1):e0191094. doi: 10.1371/journal.pone.0191094 (PMC5764345; doi:10.1371/journal.pone.0191094)
Supplement: S3 File — (DOCX) [file pone.0191094.s003.docx]

**Summary of headache characteristics in the Migraine and Probable Migraine participants in Survey 1.**

|  | | Migraine (*N* = 74) | Probable Migraine (*N* = 144) | Test for group differences |
| --- | --- | --- | --- | --- |
| Duration of headache (mean years [*SD*]) | | 8.86 (6.79) | 5.22 (4.47) | *t*(106.43) = 4.17, *p* < 0.001, *d* = 0.68 |
| Headache characteristic (*N* [%]) | |  |  |  |
|  | Unilateral pain | 42 (56.76) | 58 (40.28) | *χ*^2^(1) = 5.35, *p* = 0.02, *φ* = 0.16 |
|  | Pulsating pain | 64 (86.49) | 107 (74.31) | *χ*^2^(1) = 4.29, *p* = 0.04, *φ* = 0.14 |
|  | Moderate or severe intensity | 58 (78.38) | 48 (33.33) | *χ*^2^(1) = 39.70, *p* < 0.001, *φ* = 0.43 |
|  | Aggravation by physical activity | 51 (68.92) | 53 (36.81) | *χ*^2^(1) = 20.21, *p* < 0.001, *φ* = 0.30 |
| Accompanying symptom (*N* [%]) | |  |  |  |
|  | Nausea and vomiting | 31 (41.89) | 28 (19.44) | *χ*^2^(1) = 12.48, *p* < 0.001, *φ* = 0.24 |
|  | Photophobia and phonophobia | 62 (83.78) | 53 (36.81) | *χ*^2^(1) = 43.28, *p* < 0.001, *φ* = 0.45 |
|  | Visual aura | 12 (16.22) | 12 (8.33) | *χ*^2^(1) = 3.10, *p* = 0.08, *φ* = 0.12 |

*N*: number; *SD*: standard deviation.


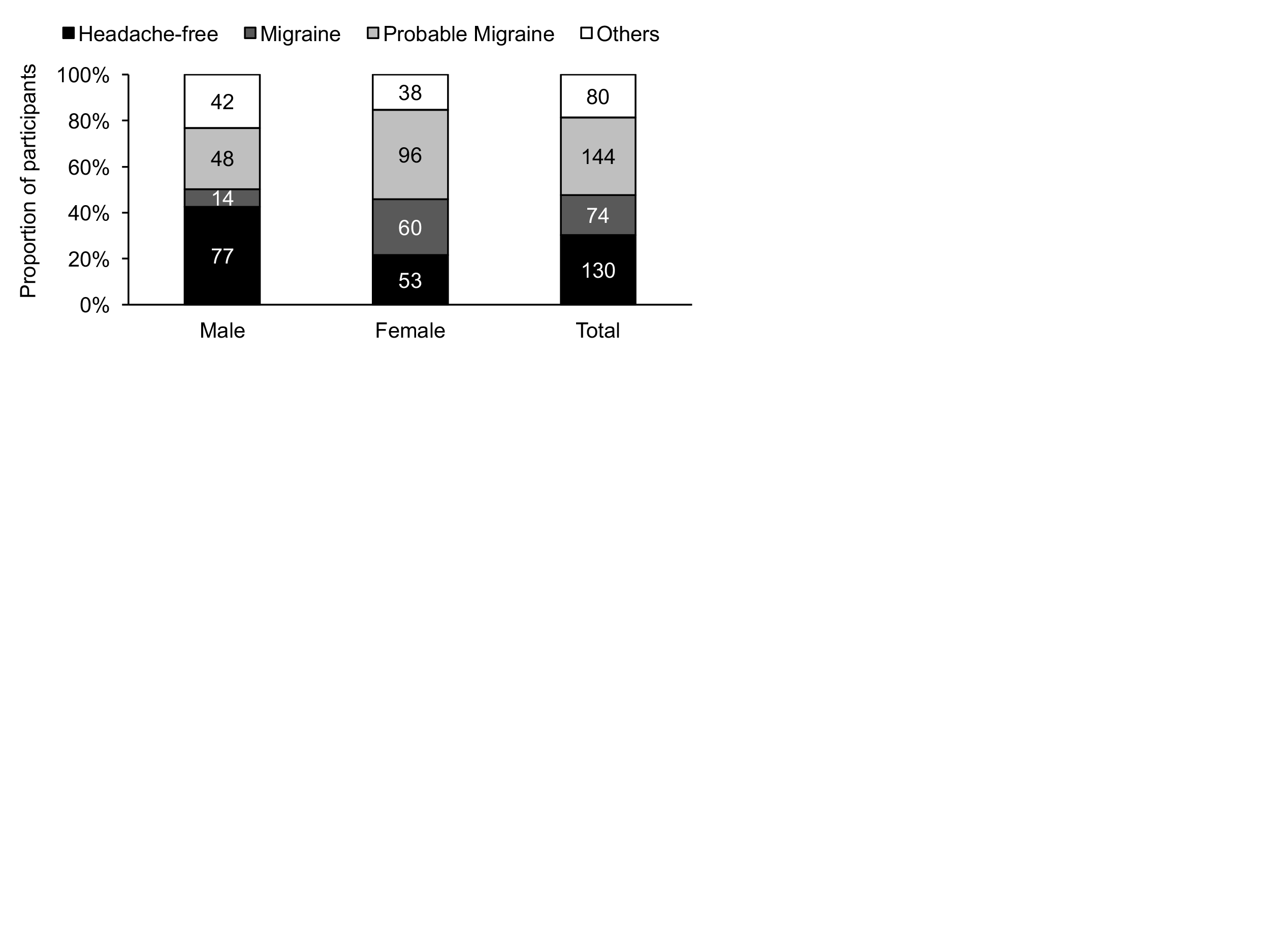


**Sex differences in primary headache prevalence in Survey 1.** Values in stacked bars denote the number of participants in each category.
