# Supplementary material for: Development of the Japanese version of the Visual Discomfort Scale
Source: PLoS One. 2018 Jan 11;13(1):e0191094. doi: 10.1371/journal.pone.0191094 (PMC5764345; doi:10.1371/journal.pone.0191094)
Supplement: S4 File — (DOCX) [file pone.0191094.s004.docx]

**Rasch statistics of the Japanese version of the Visual Discomfort Scale for 418 persons without minimum extreme, which consisted of ten participants who scored zero.**

|  | Mean | *SD* | Min | Max |
| --- | --- | --- | --- | --- |
| Total score | 12.89 | 9.54 | 1 | 57 |
| Location (standard error) | -1.90 (0.41) | 1.13 (0.18) | -4.44 (0.25) | 1.69 (1.01) |
| Infit mean-square (Zstd) | 1.00 (0.00) | 0.37 (1.10) | 0.26 (-4.00) | 2.79 (4.90) |
| Outfit mean-square (Zstd) | 0.96 (0.00) | 0.44 (1.10) | 0.31 (-3.40) | 3.85 (5.20) |
| **Real:** *RMSE* = 0.46, adjusted *SD* = 1.03, separation = 2.21, person reliability = 0.83 | | | | |
| **Model-based:** *RMSE* = 0.44, adjusted *SD* = 1.04, separation = 2.33, person reliability = 0.84 | | | | |
| Standard error of person mean = 0.06 | | | | |

*SD*: standard deviation; Zstd: z-standardized statistic; *RMSE*: root mean square error
