# Supplementary material for: Development of the Japanese version of the Visual Discomfort Scale
Source: PLoS One. 2018 Jan 11;13(1):e0191094. doi: 10.1371/journal.pone.0191094 (PMC5764345; doi:10.1371/journal.pone.0191094)
Supplement: S5 File — (DOCX) [file pone.0191094.s005.docx]

**Rasch-based psychometric properties of the 20-item version of the Japanese Visual Discomfort Scale in Survey 1.**

Items 7 and 19 of the Japanese version of the Visual Discomfort Scale (VDS) showed a misfit to the Rasch model and may be considered candidates to be removed from the scale. Analysis using the 21-item version without items 7 and 19 showed that the infit mean-square ranged from 0.75 to 1.23 (average 1.00) while the outfit mean-square ranged from 0.73 to 1.34 (average 0.96). Item 1 still showed outfit mean-square of 1.34, exceeding the criterion range [1]. We further analyzed the 20-item version without items 1, 7, and 19. The infit mean-squares ranging from 0.77 to 1.23 (average 1.04), and the outfit mean-squares ranging from 0.72 to 1.30 (average 0.97) were within the criterion range [1], suggesting that all 20 items fit well with the model.

Principal component analysis (PCA) for the 20-item version showed that 39.5% of the raw variance was explained by the measures and did not approach the cutoff of 50.0%. The eigenvalue of the unexplained variance in the first contrast was 2.21 and exceeded the cutoff of 2.00 while that of the second contrast was 1.97. These PCA results suggested the presence of multidimensionality [1]. Nevertheless, three item clusters segmented by each of first and second PCA contrasts showed strong disattenuated correlation coefficients—0.67 to 1.00 for the first contrast and 0.87 to 1.00 for the second, again implying that these item clusters virtually measured the same construct, regardless of the latent dimensions. These suggest that both the 23- and 20-item versions of the Japanese VDS showed similar dimensionality.

The 20-item version showed item reliability of 0.98 (separation 7.37) and Cronbach’s alpha of 0.90, which were comparable with those of the 23-item version. However, person reliability of 0.78 (separation 1.89) did not reach a criterion of 0.80 [2], and targeting of 2.21 was larger than 2.00, suggesting a low quality of targeting [1]. Thus, the 20-item version was inferior in person reliability and targeting to the 23-item version.

Similar to the 23-item version, several items of the 20-item version showed “mild” differential item functioning (DIF) for sex—0.68 for item 4 and 0.54 for item 5—while the others showed ignorable DIFs lower than 0.37. There were no substantial DIFs for age (lower than 0.44). Some items showed substantial DIFs for headache: item 1: *χ*^2^(3) = 8.03, *p* = 0.045; item 4: *χ*^2^(3) = 34.97, *p* < 0.001; item 5: *χ*^2^(3) = 12.62, *p* = 0.006; item 6: *χ*^2^(3) = 10.80, *p* = 0.013; item 8: *χ*^2^(3) = 15.31, *p* = 0.002. The others did not show DIF (*χ*^2^s < 6.17, *p*s > 0.10).

**References**

1. Khadka J, McAlinden C, Pesudovs K. Quality assessment of ophthalmic questionnaires: review and recommendations. Optom Vis Sci. 2013;90(8):720-44. doi:10.1097/OPX.0000000000000001. PMID:23873034

2. Linacre JM. A user's guide to WINSTEPS® MINISTEP: Rasch-model computer programs. Beaverton (OR): Winsteps.com; 2016. 719 p.
